# Factors Contributing to SARS-CoV-2 Vaccine Hesitancy of Hispanic Population in Rio Grande Valley

**DOI:** 10.3390/vaccines10081282

**Published:** 2022-08-09

**Authors:** Athina Bikaki, Michael Machiorlatti, Loren Cliff Clark, Candace A. Robledo, Ioannis A. Kakadiaris

**Affiliations:** 1 Computational Biomedicine Lab, Department of Computer Science, University of Houston, Houston, TX 77204, USA; 2 Department of Population Health and Biostatistics, University of Texas at Rio Grande Valley, Harlingen, TX 78550, USA

**Keywords:** COVID-19, decision trees, ensemble voting classification, feature selection, high-risk Hispanic population, logistic regression, multiple imputation, support vector machines, vaccine hesitancy

## Abstract

Hispanic communities have been disproportionately affected by economic disparities. These inequalities have put Hispanics at an increased risk for preventable health conditions. In addition, the CDC reports Hispanics to have 1.5× COVID-19 infection rates and low vaccination rates. This study aims to identify the driving factors for COVID-19 vaccine hesitancy of Hispanic survey participants in the Rio Grande Valley. Our analysis used machine learning methods to identify significant associations between medical, economic, and social factors impacting the uptake and willingness to receive the COVID-19 vaccine. A combination of three classification methods (i.e., logistic regression, decision trees, and support vector machines) was used to classify observations based on the value of the targeted responses received and extract a robust subset of factors. Our analysis revealed different medical, economic, and social associations that correlate to other target population groups (i.e., males and females). According to the analysis performed on males, the Matthews correlation coefficient (MCC) value was 0.972. An MCC score of 0.805 was achieved by analyzing females, while the analysis of males and females achieved 0.797. Specifically, several medical, economic factors, and sociodemographic characteristics are more prevalent in vaccine-hesitant groups, such as asthma, hypertension, mental health problems, financial strain due to COVID-19, gender, lack of health insurance plans, and limited test availability.

## 1. Introduction

COVID-19 has disproportionately affected underserved and high-risk populations, including people of different racial minority groups, underlying health conditions, and the socioeconomically disadvantaged [1]. In addition, people’s age, where people live, work, attend school, and engage in leisure activities, has been shown to be associated with health outcomes. For example, being employed in front-line service industries or living in densely populated areas may result in greater exposure to the coronavirus, making it more challenging to keep social distance [1]. Furthermore, specific access to testing, treatment, and vaccines impacts the time one receives testing and treatment [1]. COVID-19 continues to be felt across the USA. The data show a wide variation in reported vaccination rates across the United States. In a review of national studies on COVID-19 vaccination hesitancy, the overall rate of vaccine hesitancy in the general American public was 26.3% [2].

In contrast, the overall rate of COVID-19 vaccination hesitancy was higher for African Americans (41.6%) and Hispanics (30.2%) than for US Whites [2]. Researchers also found that African Americans and Hispanics were significantly more likely than non-Hispanic Whites to wait for over a year before getting vaccinated and less likely to encourage their family members to vaccinate [3]. With increasing vaccination rates in the US population, vaccine resistance and hesitancy among young African Americans may decrease as their comfort with COVID-19’s safety and efficacy increases [4]. Whereas African American and Hispanic communities are experiencing more significant adverse effects from the pandemic than other demographic groups, the overall hesitancy among Hispanics declined by approximately 9%. The rates of temporal change in vaccine hesitancy among other racial/ethnicity groups have not been shown to differ significantly from Whites [3,5]. The most significant decreases in vaccine hesitancy were observed among African Americans aged 18–24 [1].

Socioeconomic factors have also been strongly associated with COVID-19 outcomes in racial and ethnic minority populations. It is well-documented that members of underserved communities face higher rates of adverse medical conditions such as diabetes, high blood pressure, and heart disease. Most findings indicate they delayed care due to fear of contracting COVID-19 in a health care setting [6]. In addition, a positive association between the lack of a primary care physician and COVID-19 positivity among Hispanic individuals was observed [7].

COVID-19 has disproportionately impacted Hispanic/Latinx communities in the United States due to preexisting social and health disparities. The Hispanic population is the largest ethnic minority group in the United States. It constitutes 18% of the US population and 94% of the Rio Grande Valley, a four-county region in South Texas. Hospitalization rates obtained from the C.D.C. COVID-19-Associated Hospitalization Surveillance Network show that the rates are significantly elevated in Hispanic communities [2]. Several socioeconomic determinants (e.g., mistrust, low income, and financial hardship) in racial and ethnic disparities of COVID-19 vaccine hesitancy outcomes support that these factors are relevant to the prediction of hesitant individuals and should be incorporated as components of future targeted interventions [3].

In this regard, we grouped several previous studies exploring the determinants of vaccine-related hesitancy into four main categories: (i) adults in different geographical areas (e.g., urban, rural); (ii) patients with breast cancer; (iii) students; and (iv) older populations. These studies aimed to understand the reasons behind the unwillingness or undecidability of people to get vaccinated, the patterns of hesitancy, the degree of hesitation, and the characteristics of people who hesitate to be vaccinated. Regarding the diversity of geographical areas, different studies were conducted in rural and urban areas to determine the variables affecting the likelihood of refusal and indecision towards a vaccine against COVID-19 and to determine the vaccine’s acceptance for different scenarios’ effectiveness and side effects [4,5,8]. Several sociodemographic factors were associated with vaccine hesitancy, such as increased mistrust and concern regarding adverse effects, ethnicity, gender, belief that the government restrictions were too lenient, and the frequency of socializing before the pandemic [4,5,8]. Regarding COVID-19 vaccine hesitancy rates in breast cancer patients, researchers studied patients residing in Mexico [5] using univariate analysis. They discovered as important vaccine-hesitancy factors the mistrust in the health care system; the misconception that the COVID-19 vaccination is contraindicated in patients with breast cancer; not having a close acquaintance already vaccinated against COVID-19; noncompliance with prior influenza immunization; age younger than 60 years; low educational attainment; and not having an intimate acquaintance deceased from COVID-19. Regarding studies related to students, a cross-sectional qualitative survey of university students across Pakistan [8] was performed based on the assumption that vaccines are only effective if a community collectively uptakes vaccination. The researchers performed statistical analysis to determine the association between university curricula and the degree of hesitancy for the COVID-19 vaccine. They concluded that most non-medical students hesitated to obtain COVID-19 vaccines than medical students who were more willing due to their knowledge and understanding of vaccines. Factors associated with high vaccine hesitancy in a study regarding the older populations conducted a cross-sectional telephone survey on vaccine hesitancy of people older than 60 years in Bangkok [9,10] revealed that low education, a lack of confidence in the healthcare system’s ability to treat patients with COVID-19, vaccine manufacturers, being offered a vaccine from an unexpected manufacturer, and a low number of new COVID-19 cases per day were all contributing to vaccine hesitancy. Understanding previous studies’ experiences and perspectives on COVID-19 vaccine hesitancy helped us understand that vaccine hesitancy can be influenced by several socioeconomic and health factors.

Historically, a traditional statistical methodology has been used in health studies. More recently, the adoption of machine learning models in health applications has become more prominent. Much machine learning and health work have focused on processes inside the hospital or clinic. Overall, as applications of machine learning in population health develop, one of the significant challenges in health equity and fairness and assessing the external validity of the research study’s conclusions outside the context of the study [9,10]. The use of machine learning methods helps capture non-linear relationships and interactions among relevant factors, more so than traditional statistical adjustment models [9,10]. This study proposes a new methodology, using already established machine learning methods to assess the factors contributing to vaccine hesitancy. The research strategy and rationale are further described and supported by our experiments. Our results hold the potential to inform future research in this area and highlight specific opportunities using machine learning synergistically with the statistical analysis methods in the population health domain.

Our work differs from previous studies in that we have examined medical, economic, and social factors associated with vaccine hesitancy among a predominant Hispanic community sample in the Rio Grande Valley (RGV). RGV is a socio-cultural region spanning the border of Texas and Mexico and is generally bilingual in English and Spanish. RGV is at the bottom of most of the health and economic lists in the US, while Hidalgo, Cameron, and Starr counties are ranked among the poorest in Texas [11].

This study explores COVID-19 vaccine hesitancy further and examines the factors that may help better understand vaccine indecisiveness among Hispanics at the RGV. Three well-performing classifiers coupled with the recursive feature elimination (RFE) method [12] were wrapped with each base classifier to extract the most significant attributes contributing to vaccine hesitancy. Our contributions are (i) discovery of the medical, economic, and social factors (e.g., age, education, language, income, financial strain to pay for food, rent, transportation, medical care, bills, mental health issues, diabetes, and hypertension) that negatively impact Hispanic’s decisions on COVID-19 vaccination, and (ii) a new methodology based on machine learning to gain insights into the most critical factors from an appropriately designed survey instrument. Note that a similar approach that combined different well-performing models and used RFE before the classification task to identify spammers in the Twitter network resulted in improved performance compared to the evaluation of the output of a single classifier [13].

## 2. Materials and Methods

This work is part of one of the projects associated with the “Texas CEAL Consortium: Community Engagement Strategies for COVID-19: Prevention and Response in Underserved Communities in Hidalgo County”. Data were drawn from an online questionnaire where individuals over 18 years old interested in participating were asked to check a box confirming their eligibility, understanding, and consent. The questionnaire targeted people residing on the southern Texas–Mexico border in the lower Rio Grande Valley (RGV) region. The questionnaire aims to help us understand the factors contributing to vaccine hesitancy or acceptance in their community.

The COVID-19 vaccines became available to front-line health workers in December 2020. The vaccine was available to high-risk groups starting in January 2021. The vaccine was available to adults 18 and older in March 2021 [14]. In the four counties in the Rio Grande Valley covered by this study, vaccines were delivered to patients in multiple modes: vaccination events (large and small) organized by local hospital systems, safety net clinics, school districts, and county health departments; by primary care providers in private practice; by appointment at clinics; and by appointment at large national pharmacy chains and local pharmacies. The COVID-19 vaccines were administered to patients free of charge. Officially, neither was required to receive a vaccine. However, pharmacies routinely asked for proof of insurance to submit claims for reimbursement for administering the vaccines. Policies on providing proof of insurance and/or residency vary by the organization. If a person could not produce proof of insurance, the person seeking the vaccine should not be turned away. However, there were anecdotal reports of this occasionally happening in the community.

RGV is a four-county region spanning the border of Texas and Mexico. According to 2021 demographic reports, the RGV makes up 5% of the Texas population and is primarily Hispanic/Latino (94%). In addition, 56% of the population is younger than 34 years old, with 31% having limited English proficiency. During the COVID-19 pandemic, 10% of COVID-19-related deaths across the state were reported from the RGV region [15]. Although participants were drawn from the RGV at large, most respondents came from Hidalgo County.

### 2.1. Data Collection

Data were collected by asking participants to complete the surveys in four phases: baseline, 30-day, 60-day, and 90-day follow-ups. The data collection period spanned from May 2021 to December 2021. The surveys were distributed asynchronously, meaning participants could finish their 90-day follow-up while others might be completing their baseline survey. Since the baseline survey did not ask the date that a participant received a vaccine if they reported receiving one, there is no way to determine how long a person was hesitant before seeking and obtaining a COVID-19 vaccine. In addition, this paper does not examine any other instances of the longitudinal study, such as the 30-, 60-, and 90-day follow-up surveys. An analysis of that data could provide an understanding of hesitancy.

The surveys were available in English [16] and Spanish [17]. Participants were incentivized for their participation in the study. Participants received an electronic gift card (Walmart) of 10 USD to complete the baseline survey. Our analysis was based on the baseline period, with a higher completion rate (61.4%).

Ethical Considerations: The University of Texas Rio Grande Valley Institutional Review Board for Human Subjects Protections (IRB) reviewed and approved this research. Before a respondent could access the questionnaire, they were required to give informed consent to participate in the study. Their participation was voluntary and confidential, and participants were allowed to leave the study at any point.

Sample Characteristics and Data Processing: Participants were not asked to indicate the date they received the COVID-19 vaccine. If participants completed their baseline survey between May 2021 and December 2021, we could calculate the percentage who reported receiving at least one dose of a COVID-19 vaccine. Participants who did not answer the question on vaccine hesitancy (19.3%) or had not answered any of the questions (designated NA) were excluded from our analysis. We have applied data preprocessing to address: (i) missing values, (ii) duplicate instances, (iii) further grouping of categorical values, (iv) filtering to specific sociodemographic characteristics, and (v) feature selection.

### 2.2. Responses

#### 2.2.1. Summary of Responses

Table 1 summarizes the distribution of respondents in our survey during the filtering procedure. A total of 307 participants consented to answer the survey, whereas a smaller number, 296, answered the survey without missing data. Descriptive statistics are reported for this sample in Table 2. In our analysis, we are interested in the characteristics of the population, 239, who answered the question: *How likely are you to get a COVID-19 vaccine when it becomes available*? filter on the specific demographic characteristics. We focused our analysis on Hispanic males and females residing in Hidalgo County, who are the most significant part of our population.

We have categorized responses into two groups of interest: those who are already vaccinated or are willing to get vaccinated and those who are hesitant.

#### 2.2.2. Outcome Variables

Groupwise comparisons were performed to assess vaccine hesitancy with the following question:

*How likely are you to get a COVID-19 vaccine when it becomes available?*

**Figure 1:**
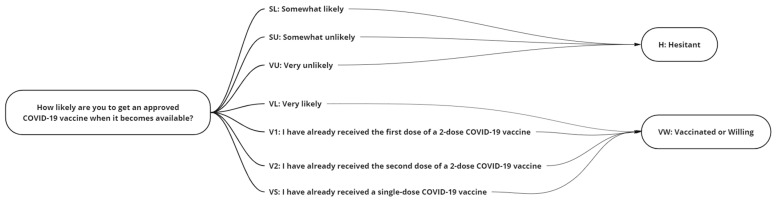
The grouping of responses to the question “How likely are you to get a COVID-19 vaccine when it becomes available?”.

The recorded responses were portioned into a two-class grouping (Figure 1) Hesitant {Somewhat Likely (SL), Somewhat Unlikely (SU), Very Unlikely (VU)}, Vaccinated or Willing {Received One Dose (V1), Received Two Doses (V2), Single Dose (VS), Very Likely (VL)} with participants who did not answer {NA} removed from the study. Having assumed that the SL respondents had switched to the vaccine-hesitant group in our case, we, therefore, grouped responses of “Somewhat likely” and “Somewhat unlikely” as vaccine hesitancy in our case, whereas “Vaccinated” and “Very likely” were grouped as vaccinated or show a willingness to get vaccinated.

#### 2.2.3. Missing Data Imputation

Missing data were present on many of the independent variables in the model. A complete listing of the variables and rate of missingness is noted in Table 3. Prior studies have shown that using thorough case analysis leads to biased results unless data are missing completely at random (MCAR), with the use of some imputation procedures providing a way to get more consistent estimates [18,19,20]. Consequently, we use imputation in this study. Before employing an imputation procedure, diagnosing the missing data patterns is essential. Using the terminology employed by [21,22], we can note the three mechanisms are MCAR, missing at random (MAR), and missing not at random (MNAR) [21,22]. MCAR implies the missing data are entirely unsystematic. In this case, deletion of missing data can be utilized and unbiased, although potentially less efficient estimates may be obtained. If data are MAR, the propensity of the data to be missing is not related to the missing data but the observed data. If the missing data are systematic and related to the missing data, which can be quantified via a missing data indicator, then it is deemed MNAR. Consequently, a missing indicator was created, and exploration with relationship to vaccine hesitancy was explored using a chi-square test or Fisher’s exact test when cell counts were low to examine if the missing at random (MAR) assumption is reasonable. No test can conclusively determine if data falls under these categories. The test was used to ascertain what is plausible and can allow us to rule out certain missing cases. All the *p*-values were greater than 0.05 except for income, where missing values in those willing to get a vaccine represented (27 of 164) 16.9% of responses while those in the unwilling was 1.7% (1 of 26). This provides evidence that a MAR assumption is reasonable. Using the findings of Collins et al. (2001) [20], we employ multiple imputation (MI) to impute missing values. They found multiple imputation (MI) was robust to MNAR in some extreme cases, even with higher rates of omission. The use of MI is further supported in this case, given our missingness rate is less than 25% and in the presence of our other covariates in logistic regression, a linear model to predict missingness indicator with c-statistic greater than 0.75 using complete cases [20]. MI is a method proposed by Rubin and Rosenbaum [23] that involves imputing m (>1) datasets, analyzing the imputed data, and then pooling the results [23,24]. To obtain an appropriate variance estimate in the pooling phase, the forecast must consider some values that are imputations. We must consider the within and between variance estimates for appropriate variance estimates. The final variance estimate, which is a combination of both, can be written as $T=\bar{U}+\left[1+\left(\frac{1}{m}\right)\right]B$, where $\bar{U}=m^{-1}\sum_{i=1}^{m}{\hat{U}}_{i}$ is the within-imputation variance and $B={\left(m-1\right)}^{-1}\sum_{i=1}^{m}{\left({\hat{Q}}_{i}-\bar{Q}\right)}^{2}$ the between imputation estimates. We note as ${\hat{Q}}_{i}$ the *i*th estimated and as $\bar{Q}\;$ the average estimate. Inside MI, one can make very stringent assumptions, such as the normality of the data, or can use more flexible approaches, where non-normality is considered. For this study, a fully conditional approach is used, noting the use of a fully conditional approach because multivariate normality is relaxed, and univariate missing models can be tailored directly to the type of variable and data type being imputed. Using *Proc MI*, a fully conditional sequential imputation procedure was used to predict missing values, given there was no discernable missing data pattern detected in the data. A value of m = 10 was used, consistent with the literature, to estimate missing data consistently [24].

#### 2.2.4. Factors

To explore how extended factors were associated with vaccine hesitancy, variables were grouped into health-related, economic, and sociodemographic questions (Table 2).

### 2.3. Analysis

#### 2.3.1. Summary Statistics

This paper aims to explore what factors are associated with vaccine hesitancy. Simple descriptive statistics are created for all variables (count and percentage) using complete cases in Table 4. Bivariate measures of association with outcome using chi-square tests for complete cases and F-tests were performed on imputed data to explore their association with vaccine hesitancy and reported in Table 4 [25,26]. As part of that goal, both parametric and machine-learning-based approaches are employed. Results are reported and discussed for both procedures. Below is a discussion of the approaches used for both procedures.

#### 2.3.2. Classification Methods

Feature selection reduces the number of features by removing unwanted and noisy features in the dataset, giving low accuracy, less comprehensibility, high computational complexity, and thus low interpretability. The total information content can be obtained from fewer unique features containing maximum discrimination information about the classes. Further, the most significant features are highly correlated with the outcome variable, whereas non-correlated features act as pure noise and introduce bias in classification accuracy calculations [27]. The feature selection method aims to identify a subset of features that can describe the input data efficiently, create a robust classification model and provide insight into the underlying process that generated the data [27]. To understand which factors contribute to our population’s decision on vaccination, we have applied feature selection in a supervised learning context.

Different feature extraction or selection techniques exist in the bibliography, which can be broadly divided into filter and wrapper approaches. In the filter approach, the feature selection method is independent of the classification model, while in the wrapper approach, it is embedded within the feature subset search. In this work, we have used an unweighted combination of the individual ranking of the logistic regression (LR), the decision trees (DT), and the support vector machines (SVM) as a stacking ensemble to gain valuable insights into the importance of the features related to vaccination responses, by wrapping these algorithms to the RFE method.

Stacking ensemble learning learns how to best combine the predictions from two or more base machine learning algorithms. Stacking harnesses the capabilities of a selection of well-performing classifiers, makes stronger predictions, and performs better than any single contributing model [28]. Furthermore, by ensembling these learners, we can aggregate the results of their specific parts of each of them.

**Figure 2:**
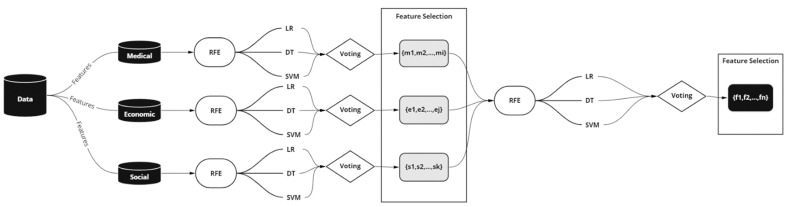
The system architecture we followed used stacking RFE wrapped with LR, DT, and SVM.

Built upon this idea, we extended the ensemble learning in our feature selection methodology and applied RFE to three classification methods: the LR, the DT, and the SVM. The classifiers, wrapped with RFE, select the most relevant features-eliminating dependent variables, resulting in stronger results and better generalization. Figure 2 depicts the system architecture. The RFE removes attributes recursively and creates a model on those remaining attributes. The model is re-fitted for each step of the elimination process. The attributes are ranked in descending order of importance via eliminating attributes with the minimum contribution. The model accuracy is used to determine which attributes significantly contribute to the target attribute prediction. This process is repeated until a specified number of features remains. The number of features to select in RFE is a hyperparameter that needs to be explored and fine-tuned, as we do not know how many features are essential in our dataset.

Learning the parameters of a prediction function is crucial in machine learning experiments. But training a model on seen data usually results in a perfect score while failing to predict yet-unseen data. This situation is called overfitting. To overcome this situation, even when evaluating different settings (hyperparameters) for a model and achieving a better generalization performance, we randomly partitioned the data into k smaller sets, “folds”, using cross-validation. In this part of our experiments, we have used the grid search method with k-fold cross-validation. The performance measured is the average of the selected scoring function in this loop. While the accuracy score is the most frequently used scoring function in classification, it is commonly used when the dataset is a balanced distribution of the predicted classes. Since we have imbalanced datasets (74.2% willing, 25.8% unwilling), and accuracy alone is not sufficient to assess the effectiveness of the model, in our experiments, we have used the Matthews correlation coefficient (MCC), a particular case of the φ coefficient, as a more appropriate measure due to class imbalance [29]. The Matthews correlation coefficient is a more reliable statistical rate that produces a high score only if the prediction obtained good results in all of the four confusion matrix categories (i.e., true positives, false negatives, true negatives, and false positives), proportionally both to the size of positive elements and the size of negative elements in the dataset [29].

#### 2.3.3. Factor Analysis (FA)

To group the importance of numerous factors to broader categories associated with medical, economic, and social, we have implemented our stacking ensemble methodology by using three traditional classifiers—LR, DT, and SVM. In addition, the features have been categorized into broader categories, which helped us evaluate the significance of each category in vaccine hesitancy. A summary of our categorization and the selected features in each category are listed in Table 2. The features were chosen not to be highly skewed, making them good candidates for our analysis. Our evaluation was performed on our target population—Hispanics residing in Hidalgo County. This study aimed to examine the factors associated with vaccine hesitancy in males and females. Furthermore, we have examined their associations with the medical, economic, and social categories and how each contributes to vaccine decisions.

Although many survey questions provide respondents with multiple answer options, our data are often limited and sparse in many of these answer options. Therefore, our machine learning models cannot effectively capture the importance of a question and its answers. A feature transformation technique such as bucketing has been applied to overcome this problem, i.e., creating new buckets based on value ranges or semantic features. To improve classification and, subsequently, the feature importance task, specific answers to selected questions have been grouped-transformed into a smaller number of groups. In addition, the survey answers were recoded to avoid the challenges of limited answers for each possibility. Therefore, specific questions were selected, and their survey answer options for that purpose, as shown in the column “Survey Answers” and the proposed transformation in column “Transformed Answers” (Table 5). In curly brackets were indicated the original categories we grouped.

#### 2.3.4. Parametric Estimation–Binary Logistic Regression

Binary logistic regression was employed to compare RFE and FA with traditional multivariable parametric analysis. A complete information linear model was employed using the same data encoded for RFE and FA. Then, the backward selection was utilized to reduce the model size to identify the variables associated with the VH variable with *p*-values < 0.15. Influencing an estimated coefficient by 10% was retained as a potential confounding variable. A more robust model considering interaction and other non-linear terms is suggested. However, a linear model was used compared to the ML methods, which utilized linear modeling [30,31]. We used *Proc Mianalyze* to get the appropriate SE and *p*-value results for the final model results, presented in Table 6. SAS 9.4 was used to perform all LR analyses.

#### 2.3.5. Model Comparisons

Two methods are used to compare the models created between ML methods and parametric estimation variables identified as significant. In the ML methods, variables ranked as important by the ensemble of RFE wrapped by the three classification methods are used. For parametric estimation, variables in the complete information linear model with *p*-values < 0.10 will be influential. In addition, a confusion table comparing model-predicted vaccine hesitancy versus actual VH status will be used to assess fit. For ML methods, a normalized MCC score > 0.5 will be considered favorable. For the binary logistic regression models, if the probability of vaccine hesitancy is higher than 0.5, then the prediction will be vaccine hesitancy ‘yes’ with ≤0.50 ‘no’. The average prediction values will be reported as both methods employed imputed data.

## 3. Results

Most participants have received the vaccine (74.2%), while the remainder reported being hesitant to receive the vaccine (25.8%). In addition, more than half the participants were females (66.8%). Finally, most of our respondents have English as their language of preference (84.2%).

As mentioned above, our analysis focused on the hesitant group {SL, SU, VU}, and our study aimed to identify the respondents’ reasons for not vaccinating. By turning the task into a binary classification problem and grouping the responses to:

- {V1, V2, VS, VL}, which corresponds to vaccinated or likely to get vaccinated, and
- {SL, SU, VU}, which corresponds to vaccine hesitancy.

In selecting {SL, SU, VU} as our target response group, we were able to classify selected factors into two distinct groups, the non-hesitant and the hesitant. The group {SL, SU, VU} was more challenging to analyze than the vaccinated or likely to get vaccinated group of {V1, V2, VS, VL} because classifiers are more sensitive to detecting the majority class and less sensitive to the minority class. This also imposes a cost for misinterpreting the features’ importance in the minority class. To address this problem, the conditional sequential imputation procedure was used to predict missing values. The augmented dataset was used to select the most important factors, where the MCC score calibrated all methods.

Having investigated the impact of several factors (Table 2) on vaccine hesitancy, our focus was on the relationship between medical, economic, and social factors. It analyzed each category separately to identify the most critical factors in the selected category. The decision was made on the factors that appeared crucial for each category—those chosen by each classifier. At the same time, the same procedure was repeated across all categories (i.e., running our classifiers to determine the most important factors). Choosing one classifier over the other would not be easy when all are well-performing. Thus, in that case, it is more appropriate to use a voting ensemble when you have two or more models that perform well on a predictive modeling task [32]. The final ranking of our attributes is a combination (unweighted voting) of the output of each model. Finally, the list of candidate features was built according to the selected method for each category separately. Males and females were investigated independently. To avoid underfitting or overfitting, fine-tuning our classifiers are required by tuning their hyperparameters and evaluating the methods using three-fold stratified cross-validation, repeated n (n = 6) times, using different data randomization in each repetition. Then, each model’s best-performing parameters for each case were selected and applied to the RFE method to get the feature ranking. The only parameter that needs to be specified in the RFE method is the selected number of features. If none is specified, it automatically selects half of the features. So, a fine-tuning of RFE was required to find the optimal number of features to choose for each model. Three experiments were carried out, i.e., evaluated together, males and females, males and females, and the results are summarized in Table 7. Each of our experiments was assessed (e.g., males, females, males, and females) by applying the three-fold stratified cross-validation method with n repetitions (n = 6) and reporting each method’s performance (Table 7). Table 7 depicts the results of our method when using the imputed dataset for males and females and males and females. MCC was selected as the calibration index for all the methods. For each classifier, we further reported the sensitivity and specificity. We also have used the same diagnostic measures to compare our methodology’s performance with the traditional binary logistic regression with backward selection when using the individual factors proposed by this method. We note that our best results are obtained using our methodology, and DT consistently outperforms the other classifiers. All our models show better predictive importance in diagnostic accuracy than the traditional methodology.

Without adjustment, age class, test availability, hypertension, and income class are associated with vaccine hesitancy with *p*-values < 0.05. After adjustment, using a backward selection linear binary logistic regression model, the factors associated with vaccine hesitancy were having a comorbid condition of hypertension and indicating a household monetary situation with insufficient funds or being forced to cut back. Those forced to cut back or not have enough funds have 9% higher odds of being vaccine hesitant. Additionally, those who indicated they have hypertension reported an 83% reduction in odds of being vaccine-hesitant versus those without.

Our analysis noted that the classification accuracy was higher when we applied our methodology to Males or Females separately. Several factors are common between the two genders, while others are more prevalent in Males or Females. Comparing the findings in Table 7 with the results in Table 6, we observe that gender contrasts with those in Table 6 and Table 7. In particular, in Table 6, gender was not statistically significant, while Table 7 shows it as an important factor.

## 4. Discussion

Our study revealed that different factors play an essential role in deciding to get vaccinated between males and females. Males seem more concerned about health issues than females during the pandemic, while females report the financial strain and the social situations they might face as more important factors. Specifically, vaccine hesitancy appears to be more prevalent in males with health issues (e.g., diabetes, hypercholesteremia, hypertension, and mental health problems). On the other hand, females tend to report more sociodemographic factors (e.g., age, education, and household size) as important towards vaccination. Then, financial strains (e.g., the ability to pay for food, medical care, credit card bills, transportation, or getting food from a food bank) negatively affect vaccination acceptance.

In conclusion, studying the survey population independently of gender, medical, and socioeconomic factors contribute to undecidability and hesitancy. Health issues such as asthma, hypertension, mental health problems, and a not very comfortable financial situation during the pandemic played an important role in vaccine undecidability. In addition, the lack of an insurance plan or medical care negatively affected their decisions. Finally, the limited availability of tests also plays an essential role in vaccination. Independently on which methods are used (traditional statistical methods or machine learning), specific factors such as hypertension, mental issues, lack of an insurance plan, and financial difficulty persist in the analysis.

The influence of variables as a confounding factor regarding vaccine hesitancy must also be considered. The variables in the study were distributed equally between willing and hesitant groups whenever possible to avoid undesired effects of confounding factors. That way, confounding factors that might arise from a skewed distribution were minimized. However, a wide range of other confounding factors can either obscure or enhance the detection of vaccine-hesitating factors that were not taken into consideration, i.e., the temporal scale of investigation may strongly influence the results of a study, the social interactions (e.g., contacts, social media), or the incentives of filling out the survey.

To maximize positive health outcomes for all, intensified and sustained efforts to dismantle inequities (e.g., income, employment, housing, education, and physical and social environment) have provided fertile ground for health inequities in Hispanic communities [33]. Therefore, it is crucial to develop a clear understanding of the critical drivers of vaccine hesitancy, develop more targeted and effective vaccine promotion interventions, and act at local, state, and national levels to improve healthcare access and economic and legal protections for immigrant communities. Moreover, understanding key health and socioeconomic determinants associated with COVID-19 mortality in geographic regions can help inform policy and enhance tailored interventions [3].

Our study also has some limitations. It is challenging to generalize our conclusions because of the small population size. Despite the size limitation, this study’s findings demonstrated our methods’ effectiveness in analyzing specific research questions related to vaccination by using the survey responses from our target population. While this was not a large sample, the findings in the present study are consistent with other studies conducted on the Hispanic population. Furthermore, machine learning methods have shown promise in clinical domains when the goal is to discover clusters in the data, such as survey analysis, and are increasingly being applied to make predictions related to population health. Finally, the modeling procedures used individual factors to predict outcomes. Data reduction and recombinant variables in some modeling procedures might yield different results when comparing a traditional statistical approach to machine-learning methods.

## 5. Conclusions

The role of machine learning in population health studies has been far less discussed and applied in the health literature. However, machine learning methods can offer insights from survey data even when our population is small. This study used the RFE method wrapped with multiple base classifiers to select relevant features and extract valuable information from survey answers related to respondents’ intent to express vaccination hesitation. Feature selection is essential because if the features chosen have high discriminating power, these features actively participate in the final class distribution and the final accuracy of the classifier.

This study shows that specific medical issues such as hypertension and mental health problems are more prevalent. Moreover, different economic reasons contribute to the Hispanics’ undecidability regarding vaccination. Additionally, sociodemographic characteristics such as gender, lack of a health insurance plan, and limited test availability are important factors that need to be examined and understood and may help remedy vaccine hesitancy. Unfortunately, SARS-CoV-2 has severely impacted their community, placing them at high risk of contracting the virus and developing severe COVID-19. However, the current study was conducted at a specific instant.

## Figures and Tables

**Table 1:** Number of Responses per category.

Class	n
Consented	307
Total with no missing data	296
Total who answered for vaccination	239
Number of Hispanics who answered for vaccination	220
Number of Hispanic Males and Females at Hidalgo who answered for vaccination	190

**Table 2:** Selected questions are grouped into medical, economic, and sociodemographic factors.

1	Medical	
	1.1.	Mental health issues (e.g., depression, anxiety, ADHD) (Y/N)
	1.2.	Hypertension (Y/N)
	1.3.	Diabetes (Y/N)
	1.4.	Asthma (Y/N)
2	Economic	
	2.1.	Income Class
	2.2.	Difficulty paying for food
	2.3.	Difficulty paying rent or mortgage
	2.4.	Difficulty paying for medical care
	2.5.	Difficulty paying for utility bills
	2.6.	Difficulty paying for transportation or car payments
	2.7.	Difficulty paying for credit card bills
	2.8.	Helping family with money due to unemployment
	2.9.	Family helping you with money due to unemployment
	2.10.	Family or friends moved in with you due to unemployment
	2.11.	Getting food from a food bank
	2.12.	Asking for payment relief for some of your bills
	2.13.	How would you describe the money situation in your household right now?
3	Social	
	3.1.	Gender
	3.2.	Age Class
	3.3.	Education Level
	3.4.	Language
	3.5.	Civil status
	3.6.	Household size
	3.7.	Insurance Status (Y/N)
	3.8.	Employment Status
	3.9.	COVID-19 tests availability

**Table 3:** Variable and rate of missingness.

Variable(s)	Rate of Missingness n (%)
Age Class	1 (0.6%)
Education Level, size of household (HH Size), Mental Health	2 (1.1%)
Hypertension, FS1, FS3, FS4, FS8, FS10, FS11, HH Money	3 (1.6%)
Insurance Status, FS2, FS5, FS7	4 (2.1%)
FS9	5 (2.6%)
Hypercholesteremia (HCL)	6 (3.2%)
FS6	9 (4.7%)
Test Availability	22 (11.6%)
Income Class	28 (14.7%)

**Table 4:** Simple descriptive statistics n (%) for all covariates (n = 190). We denote financial strain as FS, household size as HH; missing data are present on insurance status, HH Size, educational status, mental health, hypertension, HCL, income, FS-FS11, and HH Money. See Table 1 for the exact number of missing data. The complete case and imputed value are the same if no missing data are present.

Variable	Class	Overall	Complete Case			Imputed Data		
			Vaccine Hesitancy		*p*-Value	Vaccine Hesitancy		*p*-Value
			Willing	Hesitant		Willing	Hesitant	
Gender	M	63 (33.2)	52 (31.7)	11 (42.3)	0.2861	52 (31.7)	11 (42.3)	0.2861
	F	127 (66.8)	112 (68.3)	15 (57.7)		112 (68.3)	15 (57.7)	
Language	Spanish	30 (15.8)	26 (15.9)	4 (15.4)	0.9514	26 (15.9)	4 (15.4)	0.9514
	English	160 (84.2	138 (84.1)	22 (84.6)		138 (84.1)	22 (84.6)	
Age Class	18–34	79 (41.8)	68 (41.7)	11 (42.3)	0.044	69 (42.1)	11 (42.3)	0.0435
	35–54	71 (37.6)	57 (35.0)	14 (53.9)		57 (34.8)	14 (53.9)	
	55+	39 (20.6)	38 (23.3)	1 (3.9)		38 (23.2)	1 (3.9)	
Marriage Status	Widowed/Separated/Divorced/Single	94 (49.5)	84 (51.2)	10 (38.5)	0.2267	84 (51.2)	10 (38.5)	0.2267
	Married/Couple	96 (50.5)	80 (48.8)	16 (61.5)		80 (48.8)	16 (61.5)	
Insurance Status	No	52 (28)	40 (25.0)	12 (46.2)	0.0258	41 (25.2)	12 (46.2)	0.0279
	Yes	134 (72)	120 (75.0)	14 (53.9)		123 (74.8)	14 (53.9)	
Test available	Easy/Very Easy	141 (83.9)	115 (81.0)	26 (100)	0.0069	132 (80.4)	26 (100)	0.0149
	Hard/Very Hard	27 (16.1)	27 (19.0)	-		32 (19.6)	-	
HH Size	1	14 (7.5)	13 (8.0)	1 (3.9)	0.2735	13 (8.0)	1 (3.9)	0.2783
	2	48 (25.5)	44 (27.2)	4 (15.4)		44 (27.1)	4 (15.4)	
	3+	126 (67)	105 (64.8)	21 (80.8)		107 (64.9)	21 (80.8)	
Educational Status	LT HS	20 (10.6)	18 (11.0)	2 (8.0)	0.6807	18 (11.0)	3 (10.0)	0.6872
	HS/GED	56 (29.8)	46 (28.2)	10 (40.0)		46 (28.2)	10 (40.0)	
	Some College AA/AS	49 (26.1)	43 (26.4)	6 (24.0)		43 (26.3)	6 (23.1)	
	BA/BS or Higher	63 (33.5)	56 (34.4)	7 (28.0)		56 (34.4)	7 (26.9)	
Mental Health	No	151 (80.3)	126 (77.8)	25 (96.2)	0.0287	126 (77.1)	25 (96.2)	0.0247
	Yes	37 (19.7)	36 (22.2)	1 (3.9)		38 (22.9)	1 (3.9)	
Hypertension	No	133 (71.1)	111 (68.5)	22 (88.0)	0.0454	113 (68.9)	23 (88.5)	0.0399
	Yes	54 (28.9)	51 (31.5)	3 (12.0)		51 (31.1)	3 (11.5)	
HCL	No	139 (75.5)	117 (73.6)	22 (88.0)	0.1191	121 (73.8)	22 (85.8)	0.1991
	Yes	45 (24.5)	42 (26.4)	3 (12.0)		43 (26.2)	4 (14.2)	
Diabetes	No	142 (74.7)	123 (75)	19 (73.08)	0.8339	123 (75.0)	19 (73.1)	0.8339
	Yes	48 (25.3)	41 (25)	7 (26.92)		41 (25.0)	7 (26.9)	
Asthma	No	168 (88.4)	145 (88.41)	23 (88.46)	0.9945	145 (88.4)	23 (88.5)	0.9945
	Yes	22 (11.6)	19 (11.59)	3 (11.54)		19 (11.6)	3 (11.5)	
Income Class	0 to USD 39,999	97 (59.9)	85 (62.04)	12 (48)	0.0517	102 (62.2)	12 (47.3)	0.0451
	USD 40,000 to USD 69,999	44 (27.2)	32 (23.36)	12 (48)		39 (23.6)	13 (48.5)	
	USD 70,000 to USD 99,999	12 (7.4)	12 (8.76)	-		15 (8.8)	-	
	USD 100k+	9 (5.6)	8 (5.84)	1 (4)		9 (5.4)	1 (4.2)	
FS1	Somewhat hard/Not Hard	169 (90.4)	147 (89.63)	22 (95.65)	0.3595	147 (89.6)	24 (93.9)	0.4075
Pay for food	Hard/Very Hard/Cannot Afford	18 (9.6)	17 (10.37)	1 (4.35)		17 (10.4)	2 (6.2)	
FS2	Somewhat hard/Not Hard	166 (89.3)	144 (88.89)	22 (91.67)	0.6818	146 (88.7)	24 (92.3)	0.5854
Pay for rent/mortgage	Hard/Very Hard/Cannot Afford	20 (10.8)	18 (11.11)	2 (8.33)		19 (11.3)	2 (7.7)	
FS3	Somewhat hard/Not Hard	161 (86.1)	139 (85.28)	22 (91.67)	0.3982	140 (85.3)	23 (89.6)	0.5604
Pay for medical care	Hard/Very Hard/Cannot Afford	26 (13.9)	24 (14.72)	2 (8.33)		24 (14.7)	3 (10.4)	
FS4	Somewhat hard/Not Hard	160 (85.6)	138 (84.66)	22 (91.67)	0.3621	138 (84.2)	23 (88.1)	0.6373
Pay for utility bills	Hard/Very Hard/Cannot Afford	27 (14.4)	25 (15.34)	2 (8.33)		26 (15.9)	3.1 (11.9)	
FS5	Somewhat hard/Not Hard	167 (89.8)	144 (88.89)	23 (95.83)	0.2945	146 (89.0)	24 (91.9)	0.6368
Pay for transportation/car payments	Hard/Very Hard/Cannot Afford	19 (10.2)	18 (11.11)	1 (4.17)		18 (11.0)	2 (8.1)	
FS6	Somewhat hard/Not Hard	157 (86.7)	136 (86.62)	21 (87.5)	0.9062	140 (85.4)	22 (85.4)	0.6861
Pay for credit card bills	Hard/Very Hard/Cannot Afford	24 (13.3)	21 (13.38)	3 (12.5)		24 (14.6)	4 (14.6)	
FS7	No	147 (79)	127 (77.91)	20 (86.96)	0.3186	128 (78.1)	22 (85.8)	0.4111
Helping family with money due to unemployment	Yes	39 (21)	36 (22.09)	3 (13.04)		36 (22.0)	4 (14.2)	
FS8	No	167 (89.3)	145 (88.96)	22 (91.67)	0.6884	146 (89.0)	24 (91.9)	0.6454
Family helping you with money due to unemployment	Yes	20 (10.7)	18 (11.04)	2 (8.33)		18 (11.0)	2 (8.1)	
FS9	No	170 (91.9)	149 (91.41)	21 (95.45)	0.5143	150 (91.4)	24 (93.1)	0.4845
Family or friends moved in with you due to unemployment	Yes	15 (8.1)	14 (8.59)	1 (4.55)		14 (8.6)	2 (6.9)	
FS10	No	168 (89.8)	144 (88.34)	24 (100)	0.0776	144 (87.9)	26 (100)	0.0605
Getting food from a food bank	Yes	19 (10.2)	19 (11.66)	-		20 (12.1)	-	
FS11	No	170 (90.9)	147 (90.18)	23 (95.83)	0.3688	148 (90.2)	24 (91.9)	0.5960
Asking for payment relief for some of your bills	Yes	17 (9.1)	16 (9.82)	1 (4.17)		16 (9.8)	2 (8.1)	
HH Money	Have to Cut Back/Cannot Make Ends Meet	47 (25.1)	39 (23.93)	8 (33.33)	0.3213	39 (23.9)	9 (34.6)	0.2740
	Comfortable/Enough But No Extra	140 (74.9)	124 (76.07)	16 (66.67)		125 (76.1)	17 (65.4)	
Vaccine	Willing	164 (86.3)	N/A	N/A		N/A	N/A	N/A
	Hesitant	26 (13.7)	N/A	N/A		N/A	N/A	N/A

**Table 5:** Selected transformations on the answers to specific questions.

Question	Survey Answers	Transformed Answers
2.1	10.000 or less 10.000–19.999 20.000–29.999 30.000–39.999 40.000–49.999 50.000–59.999 60.000–69.999 70.000–79.999 80.000–89.999 90.000–99.999 Over 100.000	0–39.999 {1,2,3,4} 40.000–59.999 {5,6} Over 60.000 {7,8,9,10,11}
2.2, 2.3, 2.4, 2.5, 2.6, 2.7	Very hard Hard Somewhat hard Not very hard I cannot afford this anymore	Hard {1,2,5} Somewhat hard {3,4}
2.13	Comfortable with extra Enough but no extra Have to cut back Cannot make ends meet	Somewhat comfortable {1,2} Have to cut back {3} Cannot make ends meet {4}
3.1	Free text (age)	18–34 35–54 Over 55
3.2	Less than high school Some high school High school graduate or GED. Associate’s or technical degree Bachelor’s degree Graduate degree No Answer	Up to high school studies {1,2,3} Undergraduate/Graduate studies {4,5,6}
3.4	Married Widowed Separated Divorced Single, never married A member of an unmarried couple	Not married {2,3,4,5} Married {1,6}
3.5	1 2 3 4 5 6 or more	1 {1} 2 {2} 3 or more {3,4,5,6}
3.8	Very easy Easy Very hard Hard	Easy {1,2} Hard {3,4}

**Table 6:** Binary logistic regression results–backwards selection. This model correctly predicts 63.89% of hesitant or willing on average over the ten imputation models using gender, mental health, hypertension, diabetes, and response to household money. * *p* < 0.10; ** 0.01 < *p* < 0.05.

Variable	Class	Est (95% CI)	OR (95% CI)	*p*-Value
Intercept	-	1.38 (−2.14, −0.61)	-	0.0004
Gender	F v. M	−0.76 (−1.68, 0.16)	0.47 (0.19, 1.18)	0.1063
MH	Yes vs. No	−1.95 (−4.03, 0.14)	0.14 (0.02, 1.15)	0.0671 *
HT	Yes vs. No	−1.79 (−3.31, −0.28)	0.17 (0.04, 0.76)	0.0205 **
Diabetes	Yes vs. No	1.07 (−0.10, 2.25)	2.93 (0.90, 9.49)	0.0732 *
HH Money	Have to Cut Back/Cannot Make End vs. Comfortable/Enough But No Extra	1.09 (0.06, 2.12)	2.98 (1.07, 8.32)	0.0373 **

**Table 7:** Classification methods evaluation for our methodology (RFE) and traditional binary logistic regression (BLR). For each case, the common factors and best classification scores are bolded.

	Gender	Method	Factors	TP	FN	TN	FP	Sens	Spec	MCC
RFE	M + F	LR	Asthma, FS3, FS9, FS10, Gender, **HH Money**, **HT**, Insurance Status, **MH**, and Test Available	177	83	1245	395	0.681	0.759	0.665
RFE	M + F	DT	Asthma, FS3, FS9, FS10, Gender, **HH Money**, **HT**, Insurance Status, **MH**, and Test Available	250	10	1345	295	**0.962**	**0.820**	**0.797**
RFE	M + F	SVM	Asthma, FS3, FS9, FS10, Gender, **HH Money**, **HT**, Insurance Status, **MH**, and Test Available	242	18	836	804	0.931	0.510	0.652
RFE	M	DT	**Diabetes**, FS5, FS6, **FS10**, HCL, **HH Money**, **HT**, Language, **MH**, Test Available	100	10	520	0	**0.909**	**1.000**	**0.972**
RFE	F	DT	Age Class, Educational Status, FS1, FS3, FS5, FS6, FS7, FS10, HH Size, **HH Money**, **HT**, Test Available	149	1	938	182	**0.993**	**0.838**	**0.805**
BLR	M + F	LR	**Diabetes**, FS5, **HH Money**, **HT**, Insurance Status, and **MH**	150	110	1121	519	0.577	0.684	0.594
BLR	M + F	DT	**Diabetes**, FS5, **HH Money**, **HT**, Insurance Status, and **MH**	220	40	1312	328	**0.846**	**0.800**	**0.745**
BLR	M + F	SVM	**Diabetes**, FS5, **HH Money**, **HT**, Insurance Status, and **MH**	135	125	1185	455	0.519	0.723	0.590

## Abbreviations

The following abbreviations are used in this manuscript:

DT	Decision Tree
FA	Factor Analysis
LR	Logistic Regression
MAR	Missing At Random
MCAR	Missing Completely at Random
MCC	Matthews correlation coefficient
MI	Multiple Imputation
MNAR	Missing Not at Random
RGV	Rio Grande Valley
RFE	Recursive Feature Elimination
SVM	Support Vector Machine
